# FDI-6 inhibits VEGF-B expression in metastatic breast cancer: a combined in vitro and in silico study

**DOI:** 10.1007/s11030-024-10891-z

**Published:** 2024-06-09

**Authors:** Zekeriya Duzgun, Funda Demirtaş Korkmaz, Egemen Akgün

**Affiliations:** Department of Medical Biology, Faculty of Medicine, Giresun University, Giresun, Turkey

**Keywords:** Angiogenesis, FDI-6, VEGF-B, Molecular dynamic simulation, Cytotoxicity

## Abstract

Angiogenesis is the process by which new blood vessels are formed to meet the oxygen and nutrient needs of tissues. This process is vitally important in many physiological and pathological conditions such as tumor growth, metastasis, and chronic inflammation. Although the relationship of FDI-6 compound with FOXM1 protein is well known in the literature, its relationship with angiogenesis is not adequately elucidated. This study investigates the relationship of FDI-6 with angiogenesis and vascular endothelial growth factor B (VEGF-B) protein expression alterations. Furthermore, the study aims to elucidate the in silico interaction of FDI-6 with the VEGFR1 protein, a key player in initiating the angiogenic process, which is activated through its binding with VEGF-B. Our results demonstrate a significant effect of FDI-6 on cell viability. Specifically, we determined that the IC50 value of FDI-6 in HUVEC cells after 24 h of treatment is 24.2 μM, and in MDA-MB-231 cells after 24 h of application, it is 10.8 μM. These findings suggest that the cytotoxic effect of FDI-6 varies depending on the cell type. In wound healing experiments, FDI-6 significantly suppressed wound closure in MDA-MB-231 cells but did not show a similar effect in HUVEC cells. This finding suggests FDI-6 may have potential cell-type-specific effects. Molecular docking studies reveal that FDI-6 exhibits a stronger interaction with the VEGFR1 protein compared to its inhibitor, a novel interaction not previously reported in the literature. Molecular dynamic simulation results demonstrate a stable interaction between FDI-6 and VEGFR1. This interaction suggests that FDI-6 might modulate mechanisms associated with angiogenesis. Our Western blot analysis results show regulatory effects of FDI-6 on the expression of the VEGF-B protein. We encourage exploration of FDI-6 as a potential therapeutic agent in pathological processes related to angiogenesis. In conclusion, this study provides a detailed examination of the relationship between FDI-6 and both the molecular interactions and protein expressions of VEGF-B. Our findings support FDI-6 as a potential therapeutic agent in pathological processes associated with angiogenesis.

## Introduction

Angiogenesis refers to the process of new blood vessel formation, and this process is tightly controlled by various pro-angiogenic and anti-angiogenic factors [1]. The dependence of human tumors on new blood vessels for growth and metastasis underscores the importance of this process [2]. Vascular endothelial growth factor (VEGF) is one of the key regulators of pathological angiogenesis and constitutes the major endothelial cell-specific signaling pathway for pathological angiogenesis involving tumor neovascularization [3]. Inhibition of the VEGF tyrosine kinase pathway can cause tumor growth arrest or regression by inhibiting the formation of new blood vessels in growing tumors [4]. A detailed understanding of this pathway allows the development of new strategies in cancer therapy. In recent years, several therapeutic approaches to inhibit the VEGF tyrosine kinase pathway have been developed and their potential for the treatment of human cancers is being evaluated in clinical trials [5]. The critical roles of the VEGF and tyrosine kinase receptor signaling pathways in angiogenesis are discussed, along with their potential applications in cancer therapy [6].

Vascular endothelial growth factor B (VEGF-B) is a crucial regulator of angiogenesis, influencing vessel growth and stability. The VEGF-B signaling pathway primarily acts through its receptor, VEGFR-1, and co-receptor, neuropilin-1 (Nrp-1). Binding of VEGF-B to VEGFR-1 and Nrp-1 initiates a complex cascade of intracellular events. This includes the recruitment of adaptor proteins like GIPC and GAIP, potentially leading to G-protein-mediated signaling. These downstream pathways ultimately converge to promote endothelial cell survival, proliferation, migration, and the formation of new blood vessels. Further understanding of the VEGF-B signaling pathway is crucial for developing targeted therapies to modulate angiogenesis in various diseases [7].

Molecular docking has become an integral part of drug design and biochemical research. This technique simulates molecular interactions between receptors and ligands, predicting the binding modes and affinities of these interactions. This technology is widely used in computer-aided drug design as it can reduce research costs. Molecular docking can be used to predict the interactions and activities of potential drug molecules in advance, thereby accelerating the drug discovery process and achieving more effective results at lower costs [8].

Molecular dynamics (MD) simulations play a significant role in molecular biology and drug discovery. MD simulations depict how proteins, nucleic acids, and other biomolecules move and interact at the atomic level. This detailed observation allows for a better understanding of molecular structures and dynamics, providing an opportunity to better understand the structural basis of diseases. MD simulations are used to understand the interaction of drug molecules with target proteins and their biological effects, serving as an important tool in drug discovery. These simulations have made significant advancements in evaluating the efficacy and safety of drug candidates [9].

Angiogenesis plays a critical role in many physiological and pathological conditions, and the relationship between FDI-6 and angiogenesis has not yet been fully elucidated. The aim of this study is to investigate the relationship between FDI-6 and angiogenesis, examine potential therapeutic applications, and determine the effects of FDI-6 on cell viability. Additionally, we aim to determine how the cytotoxic effect of FDI-6 may vary depending on the cell type. In our study, culture and passaging procedures of Human Umbilical Vein Endothelial Cells (HUVEC) and MDA-MB-231 cell lines were performed, and Trypan Blue Staining was used to assess cell viability and count. Cytotoxicity Assay (MTT) was used to evaluate the effects of FDI-6 on cell viability. Additionally, Molecular Dynamics Simulation and Free Energy Calculations were performed to understand protein–ligand interactions. Finally, Western blotting was conducted to measure VEGF protein expression, and statistical analysis was performed to evaluate the significance between cells.

## Methods

### Cell culture

HUVEC and the adenocarcinoma cell line (MDA-MB-231) were obtained from ATCC (atcc.org). For the cells to be cultured, 10% fetal bovine serum (FBS) was added to Dulbecco’s Modified Eagle Medium (DMEM). A 1% penicillin–streptomycin solution was added to the medium to prevent possible contamination. The medium for the cells was changed 2–3 times a week, and passaging was performed when certain concentrations were reached. Cell lines were propagated in a 37C° incubator environment with 5% CO_2_. The Trypan blue test was applied to monitor the viability and number of cells cultured under appropriate conditions. FDI-6 compound was purchased from SIGMA (cat no: SML1392-5MG). VEGF-B and B-actin antibody obtained from Elabscience, Houston, TX, USA.

### Cytotoxicity test (MTT)

Cultured cells were exposed to a gradient of FDI-6 concentrations ranging from 2 to 256 µM for 24 and 48 h after seeding 5000 cells in each well. 10 μl of MTT solution was added to the cells and incubated for 3–4 h. At the end of this period, the MTT dye was removed from the cells and 100 μl of dimethyl sulfoxide (DMSO) was added to each well to dissolve the formazan crystals formed by living cells.

### Analysis of cell migration with wound healing assay

Planting was done in a 6 well plate so that there will be 1 × 106 cells in each well. After the cells adhered, a scratch was made in the vertical direction with a 1 ml pipette tip 24 h later and the cells were washed at least twice with Phosphate buffered saline (PBS). Then, Dulbecco's Modified Eagle Medium (DMEM) consisting of 2% FBS containing drug concentrations was given on the cells. After 24 h, the medium was gently removed, and the wells were washed with PBS. The image on the microscope was taken with a ×10 objective. The analysis was done with Image J software, and wound healing plugin [10, 11]. The migration area was calculated as a percentage.

### Western blot assay

LDS Sample Buffer and Sample Reducing Agent (10×) were prepared for the running of proteins on sodium dodecyl-sulfate polyacrylamide gel electrophoresis (SDS-PAGE) in each 1.5 ml eppendorf tube. The tubes were incubated at 95 °C for 5 min and then immediately placed on ice. Proteins were loaded into the wells of a 4–12% Bis–Tris Gel. The Running buffer was prepared by adding 25 ml of stock running buffer to 475 ml of distilled water and poured into the gel tank. The proteins were run at 200 V for 50 min. The protein samples run on the SDS-PAGE gel were transferred from the gel to a PVDF (0.45 um) (Polyvinylidene difluoride) membrane using a Bio-Rad Gel Transfer device in 7 min. Membranes were then incubated with 1/1500 dilutions of both VEGF-B and B-Actin antibodies. Chemiluminescent substrate (Biovision, USA) was applied to the membranes for 1 min and imaging was carried out on a Bio-Rad ChemiDoc XRS + device.

### Molecular docking calculations

Compound FDI-6 (CID: 5,175,738) was obtained from pubchem databank [12]. Prior to molecular docking, the energy minimization of FDI-6 was performed with Open Babel software addon obconformer [13]. The "MMFF94" was chosen as the energy minimization algorithm [14]. 250 conformers were generated, and the 5000-step minimization process was performed using the conjugate gradient method. The convergence criterion was set to 1e-6. X-ray structure of the angiogenic protein VEGFR1 was obtained from rcsb.org (pdb: 3HNG)(rcsb.org). Autodock Vina algorithm was used to calculate the affinity of FDI-6 compound and native VEGFR1 inhibitor N-(4-chlorophenyl)-2-((pyridin-4-ylmethyl)amino)benzamide in terms of binding to VEGFR1 protein. Pyrx software was used for the automatic execution of operations [15, 16]. In the calculation process, "Exhaustiveness" was set to 8. The grid volume was set to 17Å^3^ (cubic Angstrom) targeting the catalytic center of the protein.

### Molecular dynamics simulation and free energy calculations

All simulations were conducted with a 2 femtosecond (fs) timestep using the “Leap Frog” integration in the GROMACS 2019 software under periodic boundary conditions (PBC) [17].

AMBER99SB-ILDN forcefield was chosen [18]. FDI-6 and VEGF1 inhibitor were parameterized according to the General Amber Force Field (GAFF) AM1-BCC method using the ACEPYPE tool [19–21]. The system was prepared using the “TIP3P” water model in the form of a “Rhombic Dodecahedron,” with the corner and cube distance of the protein–ligand complex being 1 nm, and the system was neutralized with 0.15 mM Na-Cl [22]. The MD simulation was carried out in 4 stages. These stages consisted of energy minimization, NVT, NPT, and MD production parts. In the first stage, energy minimization was performed according to the Steepest Descent algorithm for maximum 50,000 steps. The maximum force difference was applied as 10 kj/mol/nm. In the NVT stage, all bonds and atomic positions were fixed with the LINCS constraint algorithm at a force of 1,000 kj/mol, and a 300 picasecond (ps) simulation was performed at 2 fs intervals [23]. The pressure coupling algorithm was set to off. In the NPT stage, only bonds were fixed with the LINCS constraint algorithm, atomic positions restrictions were released. The Verlet scheme was used as the cut-off scheme, the Nose–Hoover algorithm was used as the temperature coupling algorithm, and the temperature was set to 310 K [24]. The Parrinello-Rahman algorithm, which has isothermal compressibility, was preferred as the pressure coupling algorithm, and the pressure was set to 1 atmosphere [25]. Unlike the NPT stage, the constraint algorithm was removed, and a 100 ns (ns) molecular dynamics simulation was performed. The “Particle-mesh-Ewald” was used as the long-range electrostatics algorithm [26]. Van Der Waals and short-range electrostatic interactions were truncated at 10 Å. Binding free energies were calculated using the MM/PBSA method. The free energies of enzyme-ligand interactions were calculated via g_mmpbsa tool [27]. During the final 10 ns of the 100 ns MD simulation, 100 measurements were taken at intervals of 100 ps to calculate these energies. The dielectric constant of the solute was 2, and the solvent dielectric constant was 80 in the vacuum electrostatic calculation. The Solvent Accessible Surface Area (SASA) was employed to approximate the non-polar contribution. All computational biological research was conducted at TÜBİTAK ULAKBİM High Performance and Grid Computing Center.

### Statistical analysis

A t-test was performed using GraphPad 8.0 software to determine the significance between the cells treated with the compound and the control group, and *p* < 0.05 was accepted as significant (GraphPad Software, Boston, Massachusetts USA). All tests were conducted in triplicate.

## Results and discussion

### Cytotoxicity assay

**Fig. 1:**
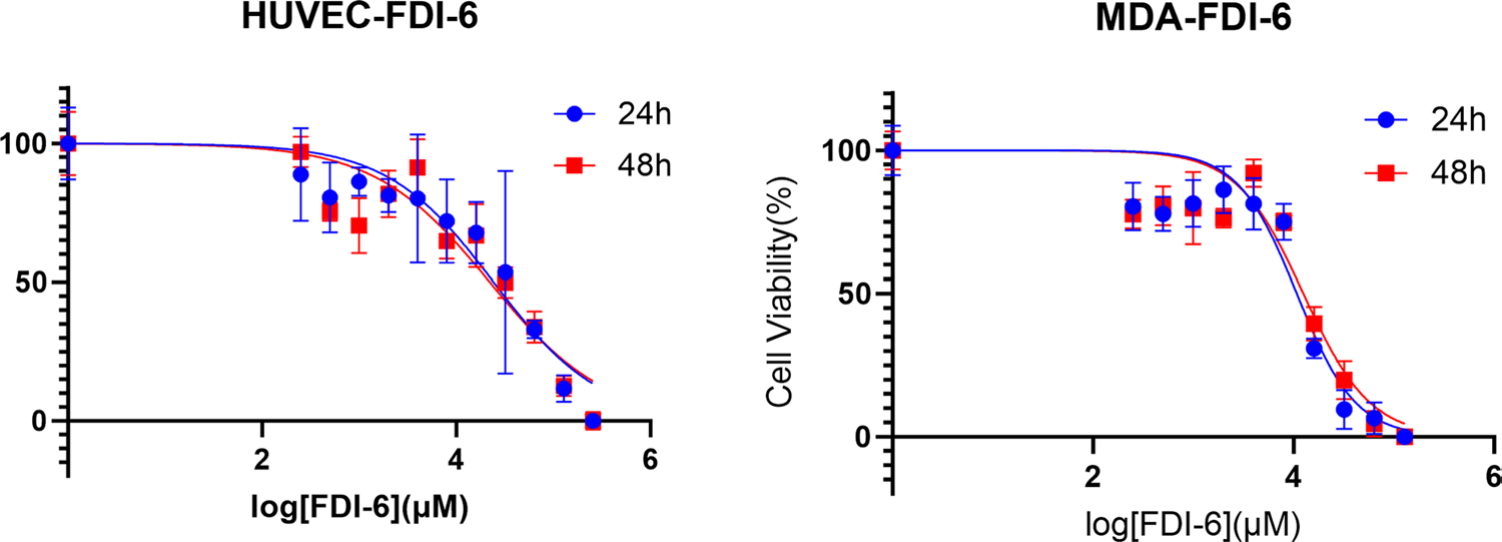
Cell viability rates of HUVEC and MDA-MB-231 cells incubated with FDI-6 at the indicated doses for 24 and 48 h

Two different cell lines were used to investigate the effects of FDI-6 on cell viability. After 24 h of treatment with FDI-6, a significant decrease in cell viability was observed in the non-malignant HUVEC cell line at a concentration of 16 µM. A significant decrease in cell viability was observed at all concentrations except 0.25 and 4 µM when FDI-6 was applied for 48 h. Therefore, the least significant concentration affecting cell viability was determined to be 8 µM. The IC50 value of FDI-6 in the HUVEC cell line was 24.2 µM at 24 h and the IC50 value was 22.1 µM at 48 h (Fig. 1). No study showing the cytotoxicity of FDI-6 in the HUVEC cell line was found in the literature. However, in the malignant MDA-MB-231 cell line, we observed an IC50 value of FDI-6 of 10.8 µM at 24 h and an IC50 value of 12.5 µM at 48 h. In addition, it was found that all concentrations of FDI-6 except 4 µM at 48 h significantly decreased cell viability at both 24 h and 48 h in the Malignant MDA-MB-231 cell line study. Studies on FDI-6 cytotoxicity on the MDA-MB-231 cell line differ. Ulhaka et al. showed the IC50 value of FDI-6 on the MDA-MB-231 cell line to be 7.33 µM, whereas in another study this value was shown to be 28.6 µM [28, 29]. Dakhili et al. synthesized ten derivatives of FDI-6 to confirm the importance of the basic halogen-binding interaction between the Arg297 residue in the FOXM1 DNA binding domain (DBD) and the 4-fluorophenyl group in forkhead domain inhibitor-6 (FDI-6) and showed an IC50 value of 31.1 µM [30]. In our study, in accordance with the dose–response curve, the IC50 (inhibition concentration) value of FDI-6 for MDA-MB-231 cells was determined to be 10.8 µM for 24 h and 12.5 µM for 48 h.

### Wound healing assay

**Fig. 2:**
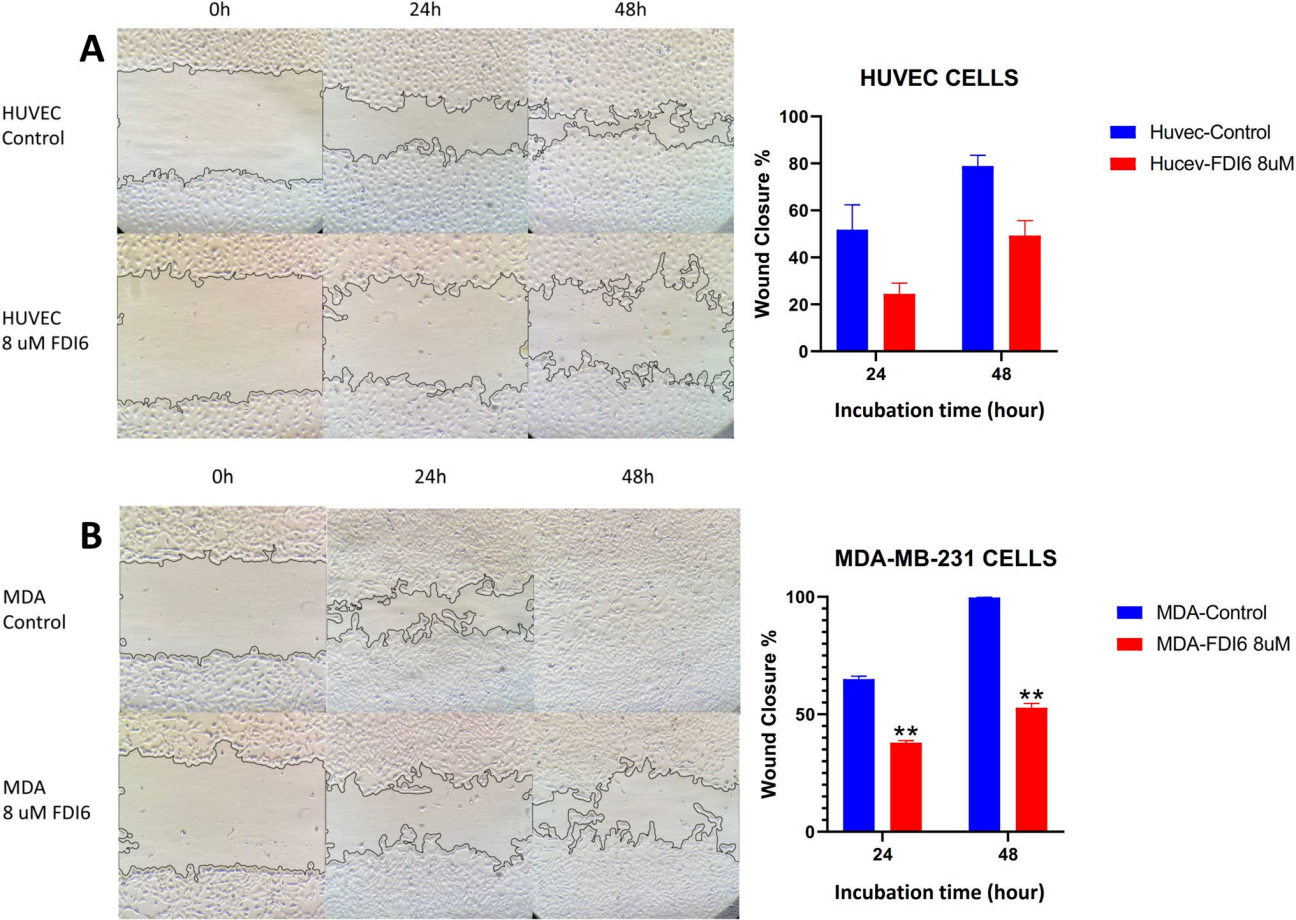
N8 µM FDI6 was shown to impair wound healing in HUVEC and MDA-MB-231 cells. At 24 and 48 h of incubation, FDI6-treated HUVEC cells (**A**) and MDA-MB-231 cells (**B**) exhibited significantly lower wound closure rates compared to control cells (*p* < 0.05). Statistical differences were calculated using Student's t-test. *p < 0.05; **p < 0.01; ***p < 0.001; ****p < 0.0001

MDA-MB-231 and HUVEC cell lines were treated with 8 µM FDI-6 and wound closure was measured at 0, 24, and 48 h. A significant increase in wound closure was observed in the MDA-MB-231 cell line treated with 8 µM FDI-6 at both 24 and 48 h. Under the same conditions in the wound closure assay, the HUVEC cell line exhibited a slower rate of closure compared to the MDA-MB-231 cell line. The study by Ulhaka et al., which examined the anticancer activity of FDI-6 on TNBC and the molecular mechanisms involved, found that FDI-6 significantly inhibited the migration ability of MDA-MB-231 cells as assessed by a wound assay (Fig. 2). In addition, FDI-6 was also shown to significantly reduce the invasion ability of TNBC cells as assessed by the transwell invasion assay [28]. In addition, various studies have investigated the effect of FDI-6 on wound healing in various cell lines and it was observed that FDI-6 significantly suppressed wound healing [31, 32].

### Molecular docking

**Fig. 3:**
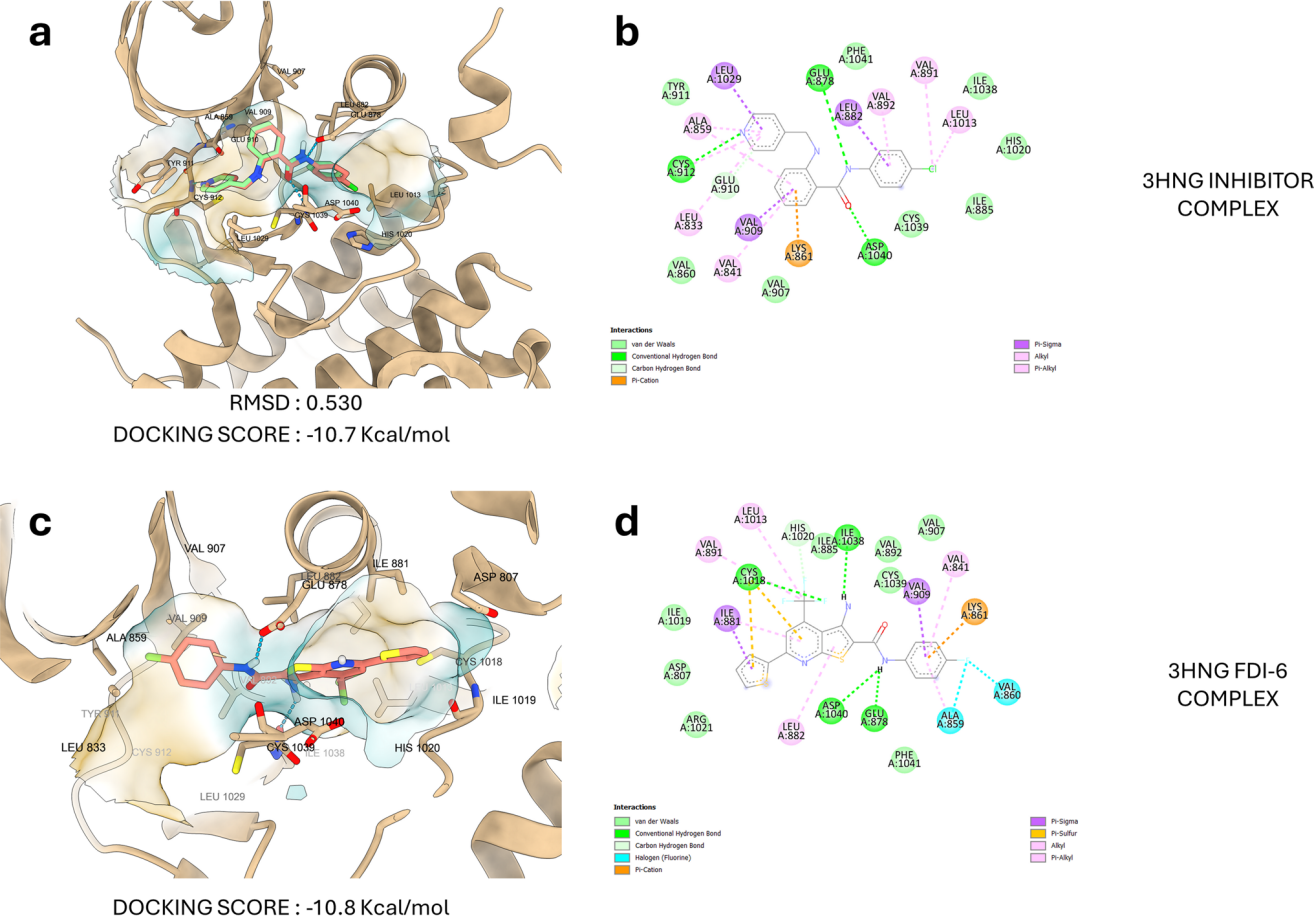
N3D conformational representation of VEGFR1 inhibitor after molecular docking (salmon color) and its original X-ray form (green) (**a**). 2D representation of the VEGFR1 inhibitor and VEGFR1 residual interactions after molecular docking (**b**). 3D conformational representation of VEGFR1 and FDI-6 compound (**c**). 2D representation of the FDI-6 compound and VEGFR1 residual interactions (**d**)

In our molecular docking study, we investigated the molecular interaction between the FDI-6 compound and the native VEGFR1 inhibitor, N-(4-chlorophenyl)-2-((pyridin-4-ylmethyl)amino)benzamide, in their binding to the VEGFR1 protein. FDI-6 was bind to VEGFR1 with a binding affinity of − 10.8 kcal/mol. On the other hand, the native VEGFR1 inhibitor, N-(4-chlorophenyl)-2-((pyridin-4-ylmethyl)amino)benzamide, was also bind to VEGFR1, but with a slightly lower binding strength of − 10.7 kcal/mol. Examining the residues involved in the interaction one by one, Glu878, Cys1018, Ile1038, and Asp1040 residues of VEGFR1 were found to form hydrogen bonds with FDI-6. Additionally, many pi-alkyl bonds were observed. Furthermore, Lys861 formed a pi-sulfur bond. Also, Ile881 and Val909 formed pi-sigma bond with FDI-6. Notably, we did not find any previous studies demonstrating the molecular interaction of FDI-6 with VEGFR1 in the literature. However, several studies have shown that FDI-6 interacts with FOXM1 at the molecular level [33, 34]. In the 3HNG crystal structure, the interaction between the native inhibitor N-(4-chlorophenyl)-2-((pyridin-4-ylmethyl)amino)benzamide and VEGFR1 was studied. It was observed that the binding interactions were similar to those with FDI-6 (Fig. 3). Like FDI-6, the native inhibitor also showed hydrogen bond interactions with Glu878 and Asp1040. However, unlike FDI-6, a hydrogen bond interaction was observed with Cys912. Both compounds were found to form a pi-sulfur bond with Lys861. The fact that FDI-6 exhibits a similar binding pattern to the native inhibitor, coupled with its slightly higher docking score, strengthens the possibility that this compound has the potential to inhibit the VEGFR1 protein.

### Molecular dynamics simulation

**Fig. 4:**
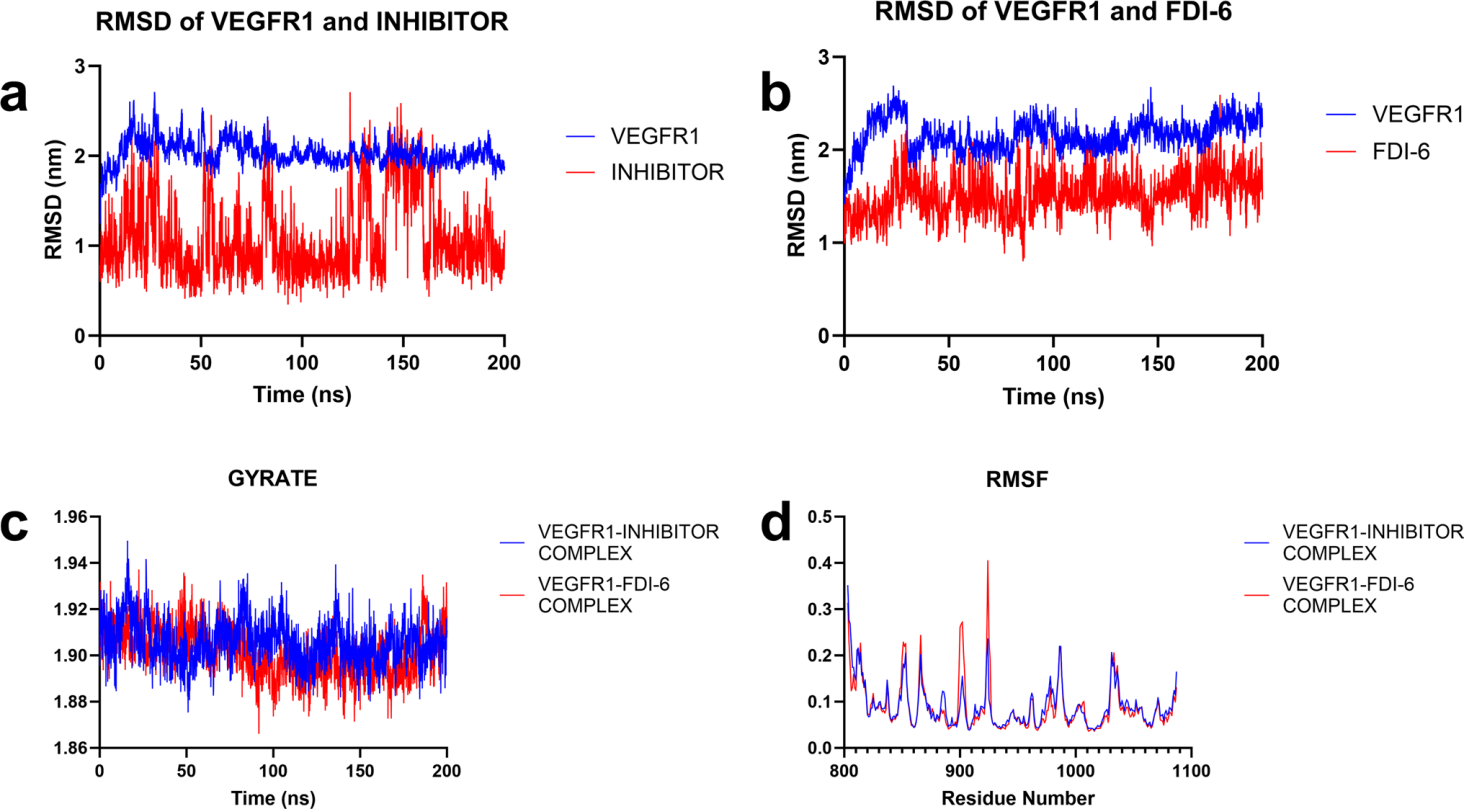
Root mean square deviation (RMSD) plot showing the stability of VEGFR1 and its inhibitor over 200 ns (**a**). RMSD plot of VEGFR1 protein and FDI6 compound over a 200 ns period (**b**). The graph plots the time in nanoseconds (ns) on the *x*-axis against the RMSD in nanometres (nm) on the y-axis. The blue line represents the VEGFR1 protein, and the red line represents the FDI6 compound. The gyrate graph measures the compactness of the VEGFR1-inhibitor complex and the VEGFR1-FDI-6 complex in 200 ns (**c**). Root mean square fluctuation (RMSF) of VEGFR1-inhibitor complex and VEGFR1 FDI-6 complex (Table 1). RMSF values provide insight into the flexibility of specific residues in the protein structure (**d**)

To determine whether the interaction of the compound FDI-6 and the native inhibitor N-(4-chlorophenyl)-2-((pyridin-4-ylmethyl)amino)benzamide with the VEGFR1 protein remains stable over time and to calculate the binding free energy of these compounds, a molecular dynamics simulation was performed. Molecular dynamics (MD) simulations have made significant advances in various disciplines such as chemistry and biophysics. This computational technique is used for chemical systems that are costly or difficult to study experimentally, for detailed characterization of biomolecular systems, to improve experimental design, and to predict relevant properties. One of the most diverse applications of MD simulations is in drug design and development [35]. In our study, the 200 ns MD simulation of the VEGFR1/FDI-6 complex showed that the protein remained stable from 200 ns on, while the FDI-6 compound showed no destabilization in the protein cavity below 2 nm (Fig. 4b). Analysis of the RMSD plot from the 200 ns MD simulation of VEGFR1 and the native inhibitor showed that, as expected, the inhibitor and protein maintained their stability throughout the simulation. However, it was observed that the inhibitor occasionally exhibited transient conformational changes, but quickly returned to its original form. (Fig. 4a). During the analysis of the 200 ns MD simulation of FDI-6 and the native inhibitor with VEGFR1, it was observed that FDI-6 formed a hydrogen bond with Glu878 at a rate of 17.24%, and interacted with Cys1039 and Asp1040 at rates of 0.05% and 1.55%, respectively, throughout the simulation. When the hydrogen bond interaction of the native inhibitor during the simulation was examined, it was found that the compound formed a hydrogen bond with Glu878 at a rate of 37.23%, and with Asp1040 at a rate of 44.13%, while it interacted with Asp238 and Cys912 at rates of 0.80% and 0.30%, respectively. These results not only indicate that Glu878 plays a significant role in binding, but also confirm that the inhibitor is more stable within the cavity compared to FDI-6.

Although there is no MD simulation in the literature that shows the interaction between VEGR1 and FDI-6, there have been several studies showing that FDI-6 interacts with FOXM1 in particular [28, 36]. Xie et al. identified XST-119, a new inhibitor for FOXM1, which plays an important role in ovarian cancer treatment, by virtual screening and experimental methods in comparison with FDI-6 and evaluated its efficacy [37]. In another study, thiazolidinedione derivatives that disrupt the interaction between FOXM1 protein and DNA binding site and inhibit the transcriptional activity of FOXM1 in triple negative breast cancer cells were designed and biologically analyzed. In the study, a compound named TFI-10 was shown to inhibit FOXM1 by an SP1-independent mechanism compared to FDI-6, and its binding mode was determined by molecular modeling and dynamic simulation studies [38].

### Free energy calculations

The MM/PBSA method is a widely used method for calculating the binding free energy of protein–ligand complexes. This method provides an effective computational cost/accuracy balance and is an important tool for drug design and discovery [39]. In our study, we calculated the binding free energy of the VEGFR1/FDI-6 and VEGFR1/inhibitor complex according to the MM/GBSA method after MD simulations. As shown in Table 2, compared to the VEGFR1 inhibitor N-(4-chlorophenyl)-2-((pyridin-4-ylmethyl)amino)benzamide, FDI-6 was found to interact with a lower van der Waals energy of − 52.15 kcal/mol. While the EGB and G SOLV values of the VEGFR1 inhibitor negatively affected the molecular interaction with 32.04 and 25.66 kcal/mol, respectively, these values in FDI-6 increased the effectiveness of the interaction with − 2.02 and − 8.91 kcal/mol, respectively. However, it was observed that the electrostatic effect contribution to the FDI-6 VEGFR1 interaction was 20.57 kcal/mol. When the total binding free energy was examined, it was observed that the VEGFR1 inhibitor bound to VEGFR1 with − 48.44 kcal/mol, but FDI-6 bound to VEGFR1 with − 40.48 kcal/mol. This situation indicates that FDI-6 may have the potential to bind to VEGFR1, but it was observed that it was not as effective as the VEGFR1 inhibitor N-(4-chlorophenyl)-2-((pyridin-4-ylmethyl)amino)benzamide. To elaborate further, the interaction of FDI-6 with VEGFR1, despite having a lower van der Waals energy, shows that it has the potential to bind to VEGFR1. The lower EGB and G SOLV values of FDI-6 compared to the VEGFR1 inhibitor suggest that FDI-6 may be more effective in certain molecular interactions. However, the higher electrostatic contribution of FDI-6 may limit its overall efficacy. Despite these promising properties, the total binding free energy of FDI-6 is lower than that of the VEGFR1 inhibitor, indicating that FDI-6, while having the potential, may not be as effective as the VEGFR1 inhibitor in binding to VEGFR1. This highlights the complexity of the molecular interactions and the need for further research to fully understand the potential of FDI-6 as a VEGFR1 inhibitor. Information in the literature on the calculation of the free energy of FDI-6 is limited. In one study, the binding free energy of FDI-6 to FOXM1 protein was calculated as − 25.2 kcal/mol using the MM/PBSA method [30].

**Table 1:** Statistics of RMSD, RMSF, and GYRATE data showing the effects of FDI-6 and VEGFR1 inhibitor on VEGFR1 protein stability

	RMSD				GYRATE		RMSF	
	VEGFR1	Inhibitor	VEGFR1	FDI-6	VEGFR1-inhibitor complex	VEGFR1-FDI-6 COMPLEX	VEGFR1-inhibitor complex	VEGFR1-FDI-6 complex
Minimum	1.247	0.3532	1.415	0.809	1.876	1.866	0.0386	0.0364
Maximum	2.704	2.702	2.684	2.583	1.949	1.937	0.3517	0.4045
Mean	2.031	1.087	2.16	1.517	1.906	1.901	0.09087	0.08945
SD	0.1393	0.4373	0.1685	0.2333	0.009771	0.01014	0.04632	0.05424
SE of mean	0.003114	0.009778	0.003768	0.005216	0.0002184	0.0002268	0.002744	0.003213

**Table 2:** Contribution of FDI-6 VEGFR1 binding compared to VEGFR1 inhibitor by MM/GBSA energy calculations

	VEGFR1 inhibitor		FDI-6	
MM/GBSA component	Free energy	Standard	Free Energy	Standard
	(kcal/mol)	deviation	(kcal/mol)	deviation
		(kcal/mol)		(kcal/mol)
VDWAALS	− 51.36	0.85	− 52.15	1.00
EEL	− 22.74	1.18	20.57	0.61
EGB	32.04	0.58	− 2.02	0.90
ESURF	− 6.38	0.01	− 6.89	0.01
G GAS	− 74.10	1.45	− 31.57	1.17
G SOLV	25.66	0.58	− 8.91	0.90
TOTAL	− 48.44	1.56	− 40.48	1.48

VDWAALS refers to Van der Waals energy. It is a type of potential energy associated with intermolecular attraction between atoms or molecules. EEL, stands for electrostatic energy. It is the energy resulting from the interactions between charged particles. EGB, stands for Generalized Born Energy. The Generalized Born Model is an approximation to the Poisson-Boltzmann equation used to calculate the electrostatic solvation energy. ESURF, stands for Solvent Accessible Surface Energy. It is an estimate of the energy contribution due to the solvent-accessible surface area of the molecule. G GAS is the gas phase energy. It is the total energy of the system in the gas phase, which includes the internal energy of the system and the kinetic energy of the center of mass. G SOLV is the solvation energy. It is the change in energy associated with the process of transferring a solute from the gas phase to a solvent.

### Western Blot analysis

**Fig. 5:**
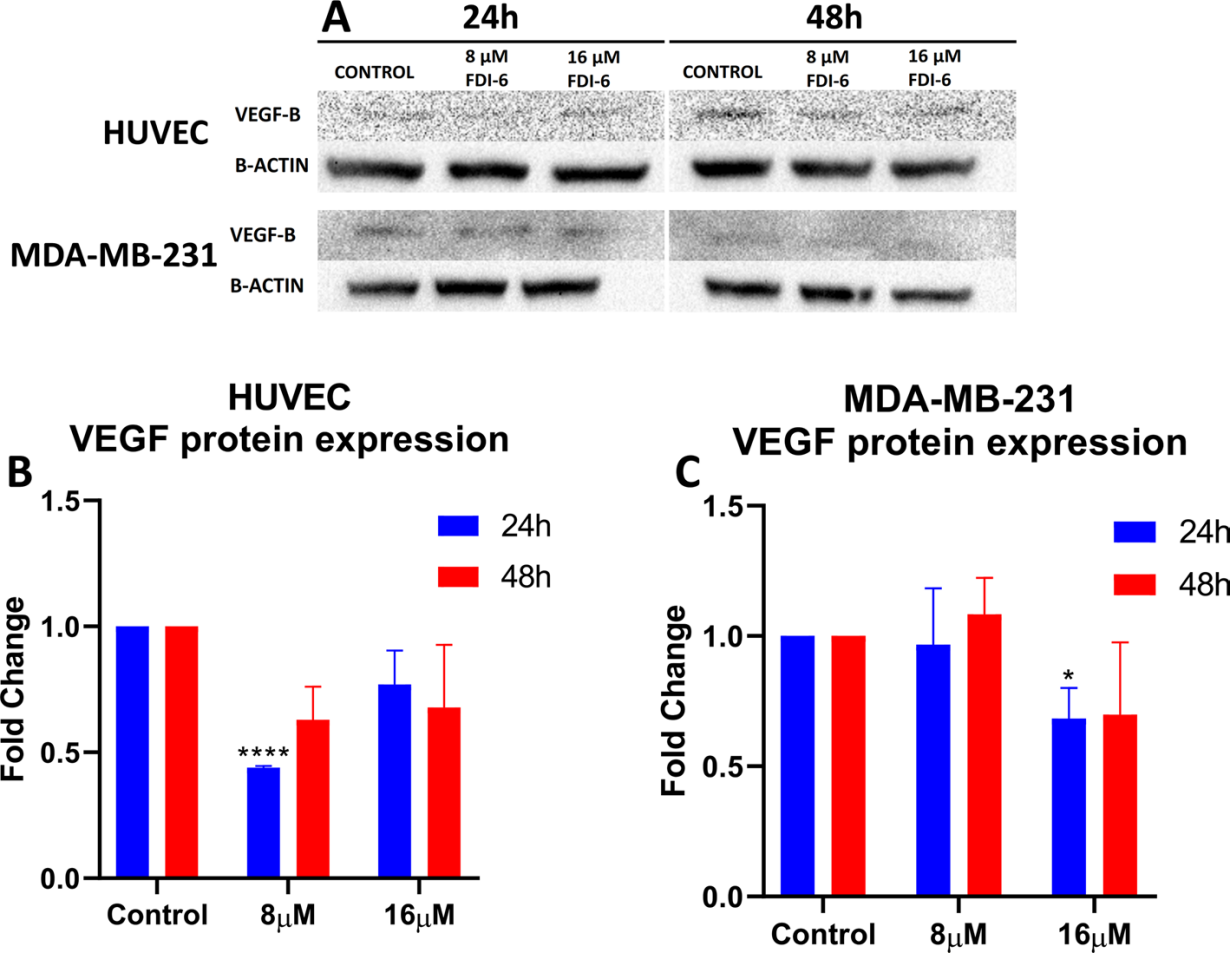
**a** Western blot image of VEGF-B and B-actin protein expression changes in HUVEC and MDA-MB-231 cell lines at 24 h and 48 h depending on 8 µM and 16 µM FDI-6 treatment. **B** B-actin normalized fold change graph of VEGF-B protein expression in HUVEC cell line due to 8 µM and 16 µM FDI-6 treatment. **C** B-actin normalized fold change graph of VEGF-B protein expression in MDA-MB-231 cell line due to 8 µM and 16 µM FDI-6 treatment

VEGF-B is involved in important biological processes such as angiogenesis, vascularization, and endothelial cell function. In this study, the effect of FDI-6 on VEGF-B protein expression in HUVEC and MDA-MB-231 cell lines was examined by Western blotting. As shown in Fig. 5, a significant change in VEGF-B protein expression was observed as FDI-6 concentration increased. Especially in the MDA-MB-231 cell line, no significant change was observed at a concentration of 8 µM, while a significant decrease in expression was observed after 24 h at a concentration of 16 µM. In the HUVEC cell line, a significant decrease was observed at 24 h at 8 µM concentration. This suggests that FDI-6 has a regulatory effect on VEGF-B. While HUVEC cell lines were derived from endothelial cells, MDA-MB-231 cell lines were derived from breast cancer cells. Comparison of VEGF-B expression profiles observed in these two different cell lines reveals cell type-specific effects of FDI-6. In conclusion, FDI-6 was observed to have potential regulatory effects on VEGF-B protein expression. These findings encourage the investigation of the use of FDI-6 as a potential therapeutic agent in pathological processes associated with VEGF-B, such as angiogenesis, tumor growth, and metastasis [40].

## Conclusion

This study provides compelling evidence for the potential of FDI-6 as a therapeutic agent in pathological processes associated with angiogenesis, particularly in metastatic breast cancer. We demonstrated that FDI-6 exhibits cytotoxic effects on both MDA-MB-231 and HUVEC cell lines, with a more cytotoxic effect observed in the malignant cell line. Furthermore, FDI-6 significantly suppressed wound closure in MDA-MB-231 cells, indicating its potential anti-migratory properties. These effects may be partially attributed to FDI-6's ability to interact with VEGFR1, a key regulator of angiogenesis, as revealed by our molecular docking and molecular dynamics simulations. Also importantly, our Western blot analysis demonstrated that FDI-6 modifies the expression of VEGF-B protein, an activator of VEGFR1 receptor protein, in a cell-type-specific manner. Our findings suggest that FDI-6 could be a promising candidate for the development of novel anti-angiogenic therapies for breast cancer and other angiogenesis-related diseases. Further in-depth investigations, including in vivo studies, are warranted to fully elucidate FDI-6's mechanism of action and explore its therapeutic potential on angiogenesis.

## Data Availability

Upon request, data to support the results of this study can be obtained from the corresponding author. During the preparation of this work, the authors used GPT4 and Gemini to identify and correct language errors. After using these tools, the authors reviewed and edited the content as needed, taking full responsibility for the content of the publication.
